# Liquid Biopsies in Cancer Diagnosis, Monitoring and Prognosis

**DOI:** 10.3390/biomedicines10112748

**Published:** 2022-10-29

**Authors:** Paola Ulivi, Stefano Indraccolo

**Affiliations:** 1 Biosciences Laboratory, IRCCS Istituto Romagnolo per lo Studio dei Tumori (IRST) “Dino Amadori”, 47014 Meldola, Italy; 2 Basic and Translational Oncology Unit, Istituto Oncologico Veneto, IOV-IRCCS, 35128 Padova, Italy; 3 Department of Surgery, Oncology and Gastroenterology, University of Padova, 35128 Padova, Italy

Liquid biopsy has emerged as new tool for detecting clinically relevant genetic alterations in cancer patients. The term liquid biopsy is comprehensive and commonly used not only to refer to molecular assays performed on cell-free DNA (cfDNA) purified from plasma but also from other body fluids, such as urine and cerebrospinal fluid, and measurements of circulating tumor cells, exosomes and cell-free RNA or other biomarkers.

We thank all the authors who contributed with their appreciated work in the Special Issue “Liquid Biopsies in Cancer Diagnosis, Monitoring and Prognosis”. Some authors highlighted the potential role of liquid biopsy in lung cancer patients. Ulivi et al. [1] reported a real-world experience on the use of liquid biopsy to investigate EGFR mutations in non-small-cell lung cancer patients. They reported that the majority of tests were performed at progression with tirosine kinase inhibitors in EGFR-mutated patients to detect the T790M mutation for potential treatment with Osimertinib. Moreover, detection of EGFR mutation in cfDNA, as an indirect index of the presence of tumor material in the blood, was associated with worse prognosis. On the same issue, Mondelo-Macia P et al. [2] discussed the role of liquid biopsy in small-cell lung cancer. For this type of cancer, a high number of circulating tumor cells (CTCs) are present, due to the high tumor growth and early disseminative capacity of the disease, leading to a potential use of this biological material for SCLC molecular analysis, although standardization of CTC isolation methods remains necessary.

The potential clinical utility of liquid biopsy in other tumor types has also been addressed. Huskova and colleagues reported that the percentage of free prostate-specific antigen (PSA), together with AMACR, PCA3 gave the best diagnostic accuracy in discriminating malignant from benign prostatic nodules, in patients with a PSA value in the grey zone (serum PSA 2.5–10 ng/mL), supporting a combined analysis of urine and blood tests could provide an accurate indication for prostate biopsy [3].

In head and neck adenoid-cystic carcinoma, the pretherapeutic serum albumin levels could predict the survival outcome. In the study by Friedl M et al., the authors performed a retrospective analysis on patients with head and neck adenoid-cystic carcinoma with available albumin levels, and they found that survival was shorter in the low-albumin group. The difference between the low- and high-albumin groups in terms of survival was significant in terms of disease-free survival, albeit not in terms of overall survival [4].

In recent years, we assisted in a substantial improvement in the technologies available for liquid biopsy. One of the established and most sensitive methodologies for the detection of actionable mutations in liquid biopsy is droplet digital PCR (ddPCR). Palacin-Aliana I et al., in their review, discussed the clinical potentiality of ddPCR to detect the most common genetic alterations in different tumor types [5].

Extracellular vesicles (EVs), and exosomes in particular, represent attractive biomarkers for liquid biopsy. However, no standardized methods are available for their isolation and characterization. Kim H et al. described a magnetic-bead-based ion exchange platform for the isolation of exosomes, called ExoCAS-2. They demonstrated a high performance of their system in comparison to other commercially available kits [6]. In the same field, Foroni C et al. described a novel EV isolation protocol to optimize the evaluation of androgen receptor (AR) gene alterations in castration-resistant prostate cancer. They demonstrated that immuno-affinity enrichment prior to RNA isolation permitted them to significantly improve the detection of AR-V7, with a better stratification between responder and non-responder patients [7].

Finally, Shueng and colleagues, in their study, described an approach aimed to increase the capacity of tumor marker detection in body fluids. Endogenous tumor blood biomarkers suffer from low concentration and from the fact that it is not possible to define the localization of biomarker production. The authors proposed an approach with the use of exogenous Sec-miR to try to solve these problems by introducing it into the cells via a non-viral episomal microcircle [8].

In conclusion, articles published in this Special Issue underscore some emerging trends in the field, including the search for technological break-throughs along with progressive inclusion of liquid-biopsy-based tests in clinical protocols involving cancer patients.
